# Measuring the Impact of a Moving Target: Towards a Dynamic Framework for Evaluating Collaborative Adaptive Interactive Technologies

**DOI:** 10.2196/jmir.1058

**Published:** 2009-06-18

**Authors:** Laura O’Grady, Holly Witteman, Jacqueline L Bender, Sara Urowitz, David Wiljer, Alejandro R Jadad

**Affiliations:** ^8^Department of AnesthesiaUniversity of TorontoTorontoONCanada; ^7^Department of Radiation OncologyUniversity of TorontoTorontoONCanada; ^6^Princess Margaret HospitalUniversity Health NetworkTorontoONCanada; ^5^Dalla Lana School of Public HealthUniversity of TorontoTorontoONCanada; ^4^Health Care, Technology and PlaceUniversity of TorontoTorontoONCanada; ^3^Mechanical and Industrial EngineeringUniversity of TorontoTorontoONCanada; ^2^Health Policy, Management and EvaluationUniversity of TorontoTorontoONCanada; ^1^Centre for Global eHealth InnovationUniversity Health NetworkTorontoONCanada

**Keywords:** Evaluation, framework, Internet, eHealth, consumer health information

## Abstract

**Background:**

Website evaluation is a key issue for researchers, organizations, and others responsible for designing, maintaining, endorsing, approving, and/or assessing the use and impact of interventions designed to influence health and health services. Traditionally, these evaluations have included elements such as content credibility, interface usability, and overall design aesthetics. With the emergence of collaborative, adaptive, and interactive ("Web 2.0") technologies such as wikis and other forms of social networking applications, these metrics may no longer be sufficient to adequately assess the quality, use or impact of a health website. Collaborative, adaptive, interactive applications support different ways for people to interact with health information on the Web, including the potential for increased user participation in the design, creation, and maintenance of such sites.

**Objective:**

We propose a framework that addresses how to evaluate collaborative, adaptive, and interactive applications.

**Methods:**

In this paper, we conducted a comprehensive review of a variety of databases using terminology related to this area.

**Results:**

We present a review of evaluation frameworks and also propose a framework that incorporates collaborative, adaptive, and interactive technologies, grounded in evaluation theory.

**Conclusion:**

This framework can be applied by researchers who wish to compare Web-based interventions, non-profit organizations, and clinical groups who aim to provide health information and support about a particular health concern via the Web, and decisions about funding grants by agencies interested in the role of social networks and collaborative, adaptive, and interactive technologies technologies to improve health and the health system.

## Introduction

Most of the publications on the evaluation of Web-based health applications focus on sites designed to provide health information to patients, their caregivers, or health professionals [1-3]. As technology changes, new challenges related to its evaluation emerge [1]. This is particularly relevant to collaborative, adaptive, and interactive technologies. We define collaborative, adaptive, and interactive technologies as technologies that (1) facilitate collaboration among users in traditional or novel ways, (2) support adaptation of form, function, and content according to user needs or preferences, and (3) enable users to interact with the technology via mechanisms of explicit interaction, such as purposefully sending data back and forth, and implicit interaction, such as exchange of data via sensors [4]. Collaborative, adaptive, and interactive technologies encompass many Web 2.0 applications, which have been described within a framework of (1) community, which relates to collaboration, and (2) information (re)organization, which necessarily draws on adaptation and interaction [5]. We view the concept of collaborative, adaptive, and interactive technologies as an umbrella definition and term that also encompasses the five major aspects of Web 2.0 health applications recently summarized by Eysenbach [6], namely: (1) social networking (collaborative and interactive), (2) participation (collaborative and adaptive), (3) apomediation (collaborative), (4) collaboration (collaborative), and (5) openness (adaptive and interactive). In this article we provide background information on evaluation designs for health-related websites, describe frameworks associated with evaluating them, and suggest a dynamic approach that could match the challenges associated with the evaluation of collaborative, adaptive, and interactive technologies.

## Methods

Eligible articles were identified through a search of (1) MEDLINE (1990 - Nov 2007), CINAHL (1990 - December 2007), Cochrane, PsycINFO (1990 - Nov 2007), Social Science Abstracts and Citation Index (1990 - 2008), and ERIC (1990 - Nov 2007); (2) personal collections of the authors; and (3) reference lists of relevant publications. The search strategy, developed in consultation with a medical librarian, included a string of Internet-related terms cross-matched with an evaluation framework string using Boolean operators. For example, the MEDLINE search used terms related to technology (Internet, World Wide Web, informatics, online), Web 2.0 terms (blog, wiki, podcast, tag), terms related to patients (some include consumer participation, education/non professional, consumer participation), and evaluation concepts (these include outcome, process, quantitative, for example). Please see Multimedia Appendix 1 for a complete list of the strategies and search terms used for each database. These searches were run again one year later when the article was accepted for publication (please see Multimedia Appendix 2).

Articles published in English in a peer-reviewed journal were deemed potentially eligible for inclusion in the review:

1. If they described a generic evaluation framework applicable to a wide range of Web-based health applications for lay members of the public; or
2. For health-specific websites, if they provided a full description of the process followed for the evaluation of such a framework that met criteria point 1.

By “generic” we meant frameworks that are applicable to sites that provide information or tools to promote decision support, social support, self-management, or self-care support.

Articles concerning evaluation of websites that provided some form of therapy or treatment such as cognitive behavioral therapy or communication with health care professionals were excluded because these sites serve a purpose that is distinctly different from the sites described above. Such sites are, in effect, a form of treatment or an extension of the clinical encounter per se, rather than a means to access information and support. As such, they have important evaluation criteria that extend beyond the website itself. We also considered out of scope those articles describing evaluation of a single formal decision aid, of diagnostic aids, or of sites designed for education of or use by medical professionals.

Three investigators (LOG, HW, JB) independently reviewed a random selection of 100 titles and abstracts from the articles that were identified through the literature search. A Single Measures Intraclass Correlation Coefficient (ICC) of 0.89 (95% CI 0.85 - 0.95) was calculated using SPSS v15.0 with a two-way mixed effects model. In the repeated search one year later, the same procedure was followed. Two of the raters had zero variance in their assessments, rendering ICC calculation not applicable. In this case, of the 100 randomly selected articles, there was agreement for 98. In both instances, the level of agreement was deemed sufficient to support independent evaluation of one-third of the total yield of the search. During independent evaluation, if the eligibility of a particular citation was judged to be questionable, the investigator included it in this initial filtering step in order to allow the other investigators to make an assessment as to whether or not it satisfied the inclusion criteria. Investigators met to review and confirm each other’s findings. Two authors (HW, JB) then reviewed the full text of all of the potentially eligible articles.

Articles were selected for inclusion in the final analysis if they described an evaluation framework applicable to Web-based, consumer-oriented health applications that could be categorized under at least two of the three core evaluation phases: (1) formative evaluation, (2) summative evaluation, and (3) outcome assessment. Within each category, parameters were organized according to these temporal phases. In order to clarify the practical differences between the phases, we describe formative evaluation as a stage of development and laboratory testing, summative evaluation as a stage of field-testing, and outcome evaluation as a stage of overall impact assessment.

For each of the 12 articles, we generated a complete list of evaluation parameters. The parameters were pooled and organized via a multidimensional card sort [7]. Using a cross-comparative analysis method we explored common themes that spanned each of the three evaluation phases.

## Results

The initial literature search yielded a total of 3304 citations, of which 41 were deemed to be potentially eligible for inclusion in the review. After reviewing the full reports, 13 articles that described evaluation frameworks met all of the inclusion criteria. Seven articles were identified from the literature search; and six were identified in personal libraries and reference lists. Two articles [8,9] were regarded as one as they discussed the same evaluation framework, producing an analysis set of 12. Of the 12 included articles, 4 described evaluation frameworks designed for Web-based, consumer-oriented health websites specifically, while 8 described evaluation frameworks designed for eHealth applications in general. Overall, 11 included elements of formative evaluation, all 12 included elements of summative evaluation, and 10 included elements of outcome evaluation. None of the articles addressed all three evaluation phases comprehensively. Flow diagrams depicting this process are available in Multimedia Appendix 3. The same steps were repeated for the second search and the flow diagram representing this process can be found in Multimedia Appendix 4.

We identified five themes that cut across the three core evaluation phases. These included an emphasis on: (1) the People affected by the website, (2) the Content of the website, (3) the Technology of the website, (4) Human-Computer Interaction between the person and the website, and (5) effects on the greater health care community, or Health Systems Integration. These themes reflect the core attributes, user-centric, context-centric, and functionality-centric, that Currie [10] advocates should be addressed in any eHealth evaluation framework.

In constructing this framework, we observed and filled in gaps relevant to collaborative, adaptive, interactive applications. For example, when evaluating applications that promote collaboration among users, we must consider interactions not only between humans and computers, but also between humans, mediated by computers. Accordingly, we refer to this theme as “Computer-Mediated Interaction” to encompass this larger scope. Articles within the review contained few to no elements corresponding to the “Content”, “Technology” and “Computer-Mediated Interaction” categories within the outcome assessment column, reflecting perhaps that general information websites, whose static content was governed mainly by webmasters, did not need to address these parameters during the outcome phases of the evaluation. However, the nature of collaborative, adaptive, interactive applications necessitates that evaluators consider and assess these parameters during the outcome phase of a project.

It has been suggested that applications that are “...interactive, user-centred, dynamic and evolving...” should have measures appropriate to these aspects [27] (page S124). Collectively, the evaluation frameworks to date demonstrated an increasing trend towards flexible, iterative evaluation designs that are user-, context-, and functionality-centric and that address multiple questions using multiple methods at each stage of the process. Similar trends in eHealth evaluation have been observed and reported by others [9,27,28]. These characteristics will be considered an issue when evaluating health sites that employ collaborative, adaptive, interactive technologies, which are considerably more fluid, dynamic, and interactive than their predecessors.

## Discussion

### A Proposed Dynamic Framework for the Evaluation of Collaborative, Adaptive, and Interactive Technologies

None of the identified frameworks matched the evaluation needs of collaborative, adaptive, and interactive technologies; therefore, we propose a new, dynamic framework in Table 1 which is described in detail below. This evaluation framework builds on our review and synthesis of existing evaluation frameworks for consumer health sites and recent descriptions of adaptive, Web-based technologies [29]. The incorporation of evaluation criteria relevant to new Web technologies addresses the gaps identified in our review and addresses the technological changes associated with collaborative, adaptive, interactive technologies, stressing their inherent social and dynamic qualities. Elements identified in the articles in our review are cited accordingly, while elements added, expanded, or adapted to reflect new areas of evaluation specific to collaborative, adaptive, and interactive technologies appear in *italics* font.

**Table 1:** Evaluation schema: collaborative, adaptive and interactive technology. Elements which were not identified in the authors' review of the literature are printed in *italics*.

	Formative Development & laboratory testing	Summative Efficacy and goal achievement	Outcome Impact assessment
People	Identification of Stakeholder Characteristics and Needs [2,7,10,11,13,14,16,17,19] *Assessment of Stakeholder Interests*	User Traits [14] Computer Proficiency [14,15],*eHealth Literacy* *Health Literacy* *Cognitive Style* *Affective Traits* User Perspectives [12,13] Intentions to Use [15] Satisfaction [7,12,13,15] *Motivation for Use[13]*	Patient Outcomes [2,10,12,13,14,15,16,18,19] Impact on Interpersonal Relationships [2,18] Patient-physician [14] *Caregiver-patient*
Content	Quality and Credibility [2,7,10,11,13,15] Utility [2,12,15] Completeness [12,15] Understandability [2,12,15] Relevance [12,15]	*Quality and Credibility[7]* Subjective Utility [16] *Level of Personalization[12]*	*Content Produced* *Form* *Nature* *Positioning of User-Generated Content*
Technology	System Robustness [18] Performance [12,15,16] Functionality and Features [7,11,12,15,16] *Security[12,15]* *Privacy* *System Interoperability* *Platforms/Portability*	Usage Statistics: Hits; Visitors; Browsers; Errors [2,10,13,14,15,16] System Reliability [7,15,18] Speed [12,18] *Positioning within Current Technology* *Standards Compliance*	*Dynamic Evolution* *Collaborative Development Models* *Open Source*
Computer-Mediated Interaction	Usability [2,7,10,12,13,16,17] Accessibility [11,16] *Sociability*[17] *Interactivity* Information Architecture [10,13]	User Perspectives on Usability[7,12,14,15,18] User Perspectives on Accessibility[15,16] Demonstrated Sociability[17] *Demonstrated Interactivity* *Collaboration* Findability [2]	*Community Development* *Evolution of Collaboration*
Health Systems Integration	Definition of Evaluation Metrics and Process [10,11] Ethics/Liability [10]	Administration [2,18,19] Service Utilization [2] Care Coordination [15,18] Patient Safety [15]	Public Impact (may include community-defined outcomes) [17,18] Cost-Effectiveness [2,7,10,12,15,16,18] Intended Effect [7] Appropriateness [15,18] Effectiveness [12,15]

### People

The category “People” contains parameters related to the individuals who are involved in using or developing the site, or who may be affected by the implementation of the site. Within this category, evaluation parameters in the formative phase consist of *Identification of Stakeholders and Stakeholder Needs* [2,8,11,12,14,15,17,18,20]. Stakeholders will necessarily include end users and may also include health care providers, funding agencies, advocacy groups, family caregivers, and people responsible for the design, development, and approval of the site. Evaluation tasks associated with this category include formal needs assessments, identification of key characteristics of potential users, and consultations with relevant stakeholders. In the summative phase, parameters within this category include: (1) *User Traits* [15], which refers to user characteristics such as computer proficiency [15,16] and demographic or disease characteristics that may affect use [2,11,14]; and (2) *User Perspectives* [13,14], which includes feedback from users or potential users regarding their *Intentions to Use* the site [16], their *Satisfaction* with the website [8,13,14,16] and their *Motivations for Use* of the site [14]. Outcome assessments for this category involve investigating the impact of the application on *Patient Outcomes*, including the psychosocial well-being, health behaviors, and physiologic outcomes of people who use the site or people for whom the site was designed [2,11,13-17,19,20]. The *Impact on Interpersonal Relationships* component [2,19] includes assessments of any change or lack of change within patient-provider relationships [15].

### People: Focus on Collaborative, Adaptive, Interactive Elements

At the formative stage, the category “People” must assess not only the informational needs of the stakeholders, but also the broader interests that will transform them from users of the site to contributors and collaborators of a dynamic enterprise transferring or generating new knowledge. We refer to this as “*Assessment of Stakeholder Interests*”. In the summative phase, the evaluative scope must expand to reflect the transition from passive learning to active participation. *Motivations for Use* of a particular site may no longer be inferred through the single or small set of purposes of a site, and may need to be evaluated more thoroughly. *User Traits* should include health literacy, health numeracy [30,31], and *eHealth Literacy* [21], which refers to how well people are able to make effective use of health information online. User traits should also include the parameter *Affective Traits* to allow for the evaluation of factors that influence social interaction such as motivation, frustration, engagement, and disengagement [29]. Previous generations of health websites could be considered as stand-alone Web destinations visited for a small range of particular purposes, such as viewing the information contained on the site or obtaining referrals to other information sites or sources. Although elements of communication (for example, Web-based message forums, newsgroups, and mailing lists) were previously available, the incorporation of collaborative, adaptive, interactive applications and features introduces a new level of complexity to health websites by expanding the functions and tasks that a user may perform at a site and by creating or reinforcing ties to other locations on the Web. Therefore, users’ *Cognitive Style* may also have an important role to play in the design of the site, given that whether or not individuals are impulsive or reflective, conceptual or inferential, thematic, or rational etc [29] will have an impact on how they experience a computer mediated-interaction. Finally, we suggest that outcome evaluations may usefully include impacts on the *Caregiver-patient* relationship, especially in cases where the application is designed to address health and life conditions involving a caregiver.

### Content

The category “Content” describes parameters related to all content on a website, including text, images, and multimedia components. In the formative phase, evaluation of content may include appraisal of content *Quality and Credibility*, such as evaluations of how accurately content represents available evidence and how well the quality is depicted [2,8,11,12,14,16]; *Utility* [2,13,16], which includes attributes such as the *Completeness* of content within the context and goals of the website program [13,16]; *Understandability*, which refers to aspects of the content such as readability statistics of text, plain language and options for translation, explanations of medical language and acronyms, choice of display formats for numerical or graphical information and clarity of images [2,13,16]; and *Relevance*, which refers to the applicability of each item of content to potential users’ health situations [13,16]. These parameters may be assessed with standardized metrics or judgment by experts and/or members of the target user population. In the summative phase, parameters within this category include *Quality and Credibility* [8], which in this phase refer to users’ perceptions of these attributes, such as whether they find the content trustworthy and believable, and *Subjective Utility* [17], or how actual users evaluate the elements of utility described in the formative phase and users’ overall assessments of the usefulness of the information on the site. Evaluation methods consist primarily of direct consultation with users via feedback mechanisms such as surveys. Finally, *Level of Personalization* [13] refers to users’ access to information that is applicable and useful to them as individuals and represents the parameter *Relevance* from the formative phase, implemented in practice.

### Content: Focus on Collaborative, Adaptive, Interactive Elements

In collaborative, adaptive, interactive applications, the potential fluidity of content presents new challenges to evaluation. The shift towards dynamic, user-generated content necessitates a change in how credibility is depicted and its subsequent assessment [33]. In this new framework there is a renewed focus on content *Quality and Credibility* (individually as in a single-author blog, or collectively as in a wiki.) With increased user-generated content, readers must be prepared to evaluate each entry, rather than each site, for its credibility. Analysis of content produced by users therefore becomes an important component of the evaluation, and the scope of *Quality* *and Credibility* evaluation expands beyond source credibility to include foci on message credibility and credibility of apomediaries [34]. In addition, adaptive and interactive features enable increases in *Level of Personalization*, expanding the scope of analysis on this element to include more detailed assessments of how personalized site content is to each user.

Outcome evaluations of collaborative, adaptive, interactive applications create entirely new requirements and avenues for evaluation. For sites that support user-generated content, *Content Produced* becomes an important output that should be investigated. Evaluations of user-generated content could involve assessment of its *Form* (narrative, numerical, and aggregated) and *Nature* (advice, opinion, personal information, and emotional support). *Positioning of User-Generated Content* may also be assessed by examining how the content provided by users is framed within the site. For example, is the user-generated content central to the site or peripheral? Is there any mechanism for feedback or dialogue between users of the site and communities of clinicians and researchers? [35].

### Technology

The category 'Technology' refers to the underlying technology used to create and run the site. The primary formative evaluation parameter discussed in the reviewed articles was *System Robustness*. This parameter includes various aspects related to performance and functionality of the technology [19]. *System Performance* refers to the quality of the infrastructure or architecture of the site including how quickly the website loads, how many concurrent users it can support, and how well the site can respond to increased requests [13,16,17]. *Functionality and Features* refer to what a user can technically do on the site and the extent to which the site's pages and external links load appropriately and without errors (eg, 404 Error indicating page not found) using a variety of different browser applications [8,12,13,16,17]. *Privacy* refers to protection of data, both stored data and data in transit, from unauthorized or unwanted disclosure, and *Security* refers to the ability to maintain control of the website and its content in the face of external threats [13,16]. In the summative phase, evaluation parameters include *Usage Statistics*, or measures of how the site is being used, such as: *Hits*, or the number of times each page is called; *Visitors*, which refers to the number of different users who visit a site and may include assessments of new and repeat visitors; *Browsers*, or the Web browsers in use by the people using the site; and incidence of *Errors*, in which visitors or software request files that do not exist or files that should exist but do not [2,11,14,15,16,17]. These metrics are commonly assessed using log file analysis [22]. Further parameters that may be evaluated at this phase include *System Reliability*, which refers to uptime and downtime, meaning the amount of time a site is available for use, as well as data corruption or loss [8,16,19], and *Speed*, which incorporates measures of performance reduction due to system load and, where appropriate, measures of database performance as a database grows [13,16]. Most of the evaluation parameters relevant to this cell may be assessed via log file analysis or Web analytics [21].

### Technology: Focus on Collaborative, Adaptive, Interactive Elements

The incorporation of new technologies into health websites serves to shift the focus of several evaluation categories. Extensive formative evaluations of *Privacy* and *Security* measures will become particularly important for Web-based applications that enable data to be shared in new ways. Traditional information websites with little to no user-generated content do not have the same critical need to consider the security of such content or the boundaries of privacy that may be challenged by people sharing sensitive, personal and identifying health information. *System Interoperability and Platforms* must be considered as additional evaluation parameters where applicable. *System Interoperability* refers to how well the site communicates with other sites and, where appropriate, how well it can be used in concert with others. This may include application programming interface (API) compatibility and data portability that allow for site integration and interactions such as mashups and syndication feeds [5]. *Platforms/Portability* refers to how well the site can be viewed and used on other devices including small-screened mobile devices, such as personal digital assistants (PDAs) and mobile phones.

In the summative phase, the dynamic nature of collaborative, adaptive, and interactive technologies prompts evaluation of the application’s *Positioning within Current Technology* and *Standards Compliance*. The former refers to the currency of the application’s technology; the latter reflects how well or poorly the site complies with Web standards and health-specific standards such as HL7. These considerations lead to outcome assessments of the *Dynamic Evolution* of the site, meaning its ability to respond to new technological and social trends. *Collaborative Development Models* refers to how the ongoing nature of the site is envisioned at the conclusion of a project. This raises the question: do *Open Source* approaches ensure the dynamic growth of platforms?

### Computer-Mediated Interaction

The category “Computer-Mediated Interaction” refers to assessments of user interactions with and via the interface. In the formative phase, evaluation parameters include *Usability* [2,8,11,13,14,17,18], which refers to how intuitive the site is for people to use [23]. In this phase, usability is typically assessed via heuristic evaluations and usability testing with sample populations of target users. Other parameters include *Accessibility* [12,17], or how well the interface is designed for people who may have barriers to use, such as vision, motor, or cognitive disabilities [24,25]; *Sociability* [18], the ability to support social interactions; and *Information Architecture* [11,14], or how well the content is organized within the site to support different information use behaviour [26]. In the summative phase, evaluation parameters within this category include user perspectives on many of the attributes assessed in the formative phase. These include: *User Perspectives on Usability* [8,13,15,16,19], which refers to actual users’ feedback on how easily and intuitively they are able to use the website; *User Perspectives on Accessibility* [16,17], which refers to feedback on barriers and enablers to use; *Demonstrated Sociability* [18], which addresses the mechanisms to promote community among users and assesses whether it is actually a sociable site now that there is a functioning community; and *Findability* [2], which refers to how well visitors who are seeking information are able to find the site. Many of the parameters in this category may be evaluated through consultations with users such as online surveys. Some, such as findability and demonstrated sociability, may also be inferred through measures of user activity.

### Computer-Mediated Interaction: Focus on Collaborative, Adaptive, Interactive Elements

At the formative stage, a shift to collaborative, adaptive, and interactive technologies expands the scope of interaction study. In the context of collaborative websites, this category is not only about interacting with the technology, but also focused on computer-assisted interactions with others. Within this category, therefore, we suggest additional focus on parameters in the formative phase such as *Sociability* [18], which refers to whether and how well the site is designed to support community interaction [36], and we propose an additional metric, *Interactivity*, or whether the interface supports adaptive, interactive human-computer behavior such as offering avenues for interface personalization.

In the summative phase, the shift to “Computer-Mediated Interaction” expands the scope of the evaluation and assessment. *Usability* and *Accessibility* testing must incorporate assessments of user interactions with changing interfaces. User-generated content, user-initiated reorganization of information, and the principle of *perpetual beta* [32], in which interfaces are presumed to be frequently changing, may all contribute to changes to the interface as seen by the user. *Demonstrated Interactivity* refers to how and whether site visitors use the interactive features of the site. Preece and colleagues [18] have done extensive work on sociability heuristics which they have thus far determined are largely community-specific. Health sites that make use of CAI tools that explicitly promote *Collaboration* as a product of *Sociability* will require evaluation of the actual collaboration that results from user interactions.

“Computer-Mediated Interaction” should also be assessed during the outcome evaluation phase and on an ongoing basis for monitoring and quality-improvement purposes. *Community Development* refers to retrospective evaluation of whether and how well the evolving site has supported community interaction. The question of how the user community has responded over time to the site is referred to as *Evolution of Collaboration*. Assessment of this parameter involves summary statistics and longitudinal analysis of evidence of collaboration within sites that incorporate collaborative features. Sites that provide mechanisms for participants to exchange information may now include blogs that allow readers to provide feedback by posting comments. If the goal of the evaluation is to assess whether community members are using the site to collaborate with each other, analyses can be conducted by examining whether the participants are exchanging messages regularly [37]. For example, a blog may attract a few comments posted by readers daily, weekly, or even monthly, and such response rates may change over time. There must be some interaction for collaboration to take place. One way to measure this is to examine the number of posted messages and their associated responses [37]. Equally important in this analysis is to review the message content and tone. A series of messages threaded together may not necessarily be a sign of collaboration; rather, it may indicate an argument. However, measuring incidents of collaboration through message postings may not be enough to prove a community is functioning effectively. A community must meet the needs of its members in order for it to be sustained. Although there may be evidence of collaboration on a large scale, some members may be posting questions and not receiving responses to their queries.

### Health Systems Integration

The category “Health Systems Integration” refers to the larger system, health processes, and society in which a health website for laypeople might be implemented. Formative evaluation parameters within this category include *Definition of Evaluation Metrics and Process* [11,12], which means whether and how well evaluation is incorporated into the design, development, and implementation of a site, and *Ethics/Liability* [11], which refers to how and whether ethical and liable issues of providing information online have been addressed within the larger health care system. Summative evaluation within this category involves assessments of how the site affects *Administration* [2,19,20], including *Service Utilization* [2], or usage rates for health care system and community services; *Care Coordination* [16,19], which refers to ways in which the site might be affecting delivery of health services; and *Patient Safety* [16], or assessments of how or whether the site is affecting patient safety indicators such as appropriate use of medications. Outcome assessment parameters within this category include *Public Impact* [18,19], which refers to any general effects that the website may have on the larger community and may include outcomes that are self-reported or defined by a particular community; *Cost-Effectiveness* [2,8,11,13,16,17,19], which refers to incremental health gain from use of the site and any associated resources; and *Intended Effect* [8], which is a context-specific assessment that will vary depending on the goals of the project and which includes *Appropriateness* [16,19], or overall observed suitability of the site as a means to achieve those goals, and *Effectiveness* [13,16], which refers to how well the site achieved its intended goals.

### Health Systems Integration: Focus on Collaborative, Adaptive, Interactive Elements

Evaluation of the integration of information technologies and their processes into the larger health system has been well-covered in previous frameworks. It is worth noting, however, that due to their expanded capabilities, collaborative, adaptive, and interactive technologies may offer both greater benefit and greater unintended consequences in this area [38]. It remains to be seen whether these potentials are actualized.

### Conclusion

We have presented an evaluation framework that proposes formative, summative, and outcome evaluation indicators for five themes of Collaborative, Adaptive, and Interactive applications: People, Content, Technology, Computer-Mediated Interaction, and Health Systems Integration. The increased use of collaborative, adaptive, and interactive technologies in health care and other fields underscores the importance of their evaluations. We need to determine whether something is effective before it can be of value. Collaborative, adaptive, interactive technologies are becoming pervasive and rapidly becoming an integral part of society. In record time, resources promoting collaboration such as Wikipedia, Facebook, MySpace, YouTube, and Orkut have joined the ranks of the most widely used online services in the world. With their vertiginous ascent, they have heralded an era in which the public can wield enormous power to create and share knowledge, to communicate with people and machines, and to find and evaluate services with unprecedented levels of freedom.

Although at a slightly slower pace, health-specific collaborative, adaptive, and interactive technologies are emerging, promising to transform the roles, workflows, rights, and responsibilities of all stakeholders within the system [38]. As any other set of interventions, however, collaborative, adaptive, and interactive technologies also carry the risk of causing more harm than good. It is a rare privilege to witness the emergence of a new set of powerful technologies that could have a profound and widespread effect on society. We should assume the responsibility that comes with such privilege “to look beyond the hype, and to dissect what works and what doesn’t” [39]. Thus, it is essential to conduct thoughtful, careful evaluations.

We offer this framework as a means to structure evaluations across a wide range of applications and purposes. In some cases there may not be sufficient resources to conduct an evaluation that addresses all of the components listed in this framework. In these situations, we suggest that the evaluation focus on the particular aspect(s) (People, Content, Technology, Computer-Mediated Interaction, Health Systems Integration) that are the most relevant to the objective(s) of the evaluation. Tailoring an outcome evaluation to the specific requirements of the funding organization rather than attempting to address all of the various components in Table 1 within this phase would be another means to reduce the resources required to measure all of the elements in every phase. Each of the components presented in the framework may also have uses beyond those of evaluative measures within an established program. For example, the components listed as formative evaluations, in particular the People, Content, and Technology sections, could also be used to form the components of a needs assessment or as part of a funding application, and the framework could be used to troubleshoot an under-utilized application.

Evaluation plays a critical role in high-quality design, efficient development, and effective implementation of Collaborative, Adaptive, and Interactive applications. In an era of constrained resource allocation, the adoption of robust and appropriate evaluation frameworks will help to ensure that collaborative, adaptive, and interactive technologies live up to the expectations and that they contribute to the improvement of health for all.

## Multimedia Appendix 1

Search strings for 2007 and prior

## Multimedia Appendix 2

Search string for 2008 - 2009

## Multimedia Appendix 3

QUOROM statement flow diagram for Literature Search 2008 and prior

## Multimedia Appendix 4

QUOROM statement flow diagram for Literature Search 2008 and 2009

## Abbreviations

API: application programming interface
HL7: health level 7
ICC: Intraclass Correlation Coefficient
PDA: personal digital assistant
