# Supplementary material for: Measuring the Impact of a Moving Target: Towards a Dynamic Framework for Evaluating Collaborative Adaptive Interactive Technologies
Source: J Med Internet Res. 2009 Jun 18;11(2):e20. doi: 10.2196/jmir.1058 (PMC2762807; doi:10.2196/jmir.1058)
Supplement: Supplementary file 1 [file jmir_v11i2e20_app1.pdf]

Appendix A: Search strings for 2007 and prior

Run January 5th, 2008

Medline

1. exp internet/
2. internet:.mp.
3. exp informatics/
4. exp computer-assisted instruction/
5. online.mp.
6. on-line.mp.
7. (virtual not virtual realit:).mp.
8. world wide web:.mp.
9. worldwide web:.mp.
10. www.mp.
11. web.mp.
12. web page:.mp.
13. webpage:.mp.
14. web site:.mp.
15. website:.mp.
16. exp computer communication networks/
17. portal?.mp.
18. ehealth.mp.
19. e-health.mp.
20. "web 2.0".mp.
21. semantic web?.mp.
22. blog:.mp.
23. collabor:.mp.
24. folksonom:.mp.
25. mashup:.mp.
26. pod cast:.mp.
27. podcast:.mp.
28. rss feed:.mp.
29. really simple syndicat:.mp.
30. rss syndicat:.mp.
31. (social adj2 bookmark:).mp.
32. (social adj2 book-mark:).mp.
33. (social adj2 software:).mp.
34. (sociable adj2 technolog:).mp.
35. (social adj2 technolog:).mp.
36. tag cloud:.mp.
37. tag????.mp.
38. videocast:.mp.
39. video-cast:.mp.
40. (virtual adj2 collabor:).mp.

41. vodcast:.mp.
42. vod-cast:.mp.
43. web api:.mp.
44. (web adj2 syndicat:).mp.
45. webcast:.mp.
46. web-cast:.mp.
47. web-log:.mp.
48. weblog:.mp.
49. wiki:.mp.
50. social network:.mp.
51. (social adj2 utilit:).mp.
52. or/1-51
53. exp Education, Nonprofessional/
54. exp Consumer Participation/
55. social support/
56. ((patient? or consumer? or client:) adj2 (decisionmak: or decision-mak:)).mp.
57. exp Self Care/
58. ((self adj2 manag:) or self-manag: or selfmanag:).mp.
59. or/53-58
60. exp evaluation studies as topic/
61. evaluation studies.pt.
62. "outcome and process assessment(health care)"/
63. "outcome assessment (health care)"/
64. "process assessment (health care)"/
65. randomized controlled trial.pt.
66. evaluation studies.pt.
67. qualitative research/
68. user-computer interface/
69. usability.mp.
70. useability.mp.
71. accessibility.mp.
72. sociability.mp.
73. log file?.mp.
74. readability.mp.
75. or/60-74
76. 52 and 59 and 75
77. limit 76 to (humans and english language)
78. limit 77 to yr="1990 - 2008"
79. remove duplicates from 78
80. 52 and 75
81. limit 80 to yr="1993 - 2008"
82. (patient? or consumer? or client:).mp. [mp=title, abstract, full text, caption text]
83. 52 and 82 and 75
84. ((patient? or consumer? or client:) adj2 (learn: or educat:)).mp.
85. 52 and 84 and 75

CIHHAL

Run January 5th, 2008

CINAHL - Cumulative Index to Nursing & Allied Health Literature 1982 to December Week 1 2007		
#	Search History	Results
1	exp internet/	34462
2	internet:.tw.	7037
3	exp informatics/	164365
4	exp computer-assisted instruction/	2822
5	online.tw.	5615
6	on-line.tw.	1032
7	(virtual not virtual realit:).tw.	1313
8	world wide web:.tw.	764
9	worldwide web:.tw.	33
10	www.tw.	194
11	web.tw.	8742
12	web page:.tw.	296
13	webpage:.tw.	9
14	web site:.tw.	2341
15	website:.tw.	1832
16	exp computer communication networks/	35948
17	portal?.tw.	776
18	ehealth.tw.	41
19	e-health.tw.	484
20	"web 2.0".tw.	22
21	semantic web?.tw.	26
22	blog:.tw.	138
23	collabor:.tw.	16922
24	folksonom:.tw.	2
25	mashup:.tw.	5
26	pod cast:.tw.	0
27	podcast:.tw.	44
28	rss feed:.tw.	17
29	really simple syndicat:.tw.	8
30	rss syndicat:.tw.	0

31	(social adj2 bookmark:).tw.	5
32	(social adj2 book-mark:).tw.	0
33	(social adj2 software:).tw.	14
34	(sociable adj2 technolog:).tw.	0
35	(social adj2 technolog:).tw.	67
36	tag cloud:.tw.	0
37	tag????.tw.	453
38	videocast:.tw.	1
39	video-cast:.tw.	1
40	vodcast:.tw.	1
41	vod-cast:.tw.	0
42	web api:.tw.	0
43	(web: adj2 syndicat:).tw.	1
44	webcast:.tw.	26
45	web-cast:.tw.	6
46	web-log:.tw.	319
47	weblog:.tw.	26
48	wiki:.tw.	38
49	social network:.tw.	1269
50	(social adj2 utilit:).tw.	25
51	or/1-50	185599
52	(evaluat: adj2 (framework: or frame-work:)).tw.	236
53	(informatic: adj2 evaluat:).tw.	4
54	(evaluat: adj2 guideline?).tw.	211
55	(evaluat: adj2 (process: or protocol: or method? or format? or outline?)).tw.	3323
56	or/52-55	3743
57	51 and 56	963
58	limit 57 to human [Limit not valid in: CINAHL; records were retained]	963
59	limit 58 to english	941
60	limit 59 to yr="1990 - 2008"	932
61	remove duplicates from 60	932
62	evaluation/ or product evaluation/	4602
63	exp evaluation research/ or exp formative evaluation research/ or exp summative evaluation research/	11650
64	or/62-63	15719
65	Conceptual Framework/	12660

66	64 and 65	239
67	51 and 66	62
68	61 or 67	985
69	limit 68 to yr="1990 - 2008"	984
70	limit 69 to english	982
71	remove duplicates from 70	982
72	limit 71 to journal article	963

Cochrane

Run January 5th, 2008

All EBM Reviews - Cochrane DSR, ACP Journal Club, DARE, CCTR, CMR, HTA, and NHSEED		
#	**Search History**	**Results**
1	exp internet/	392
2	internet:.mp.	1542
3	exp informatics/	16
4	informatic:.mp.	142
5	exp computer-assisted instruction/	419
6	(computer: adj2 instruction?).mp.	490
7	(computer: adj2 educat:).mp.	364
8	(computer: adj2 training?).mp.	131
9	online.mp.	836
10	on-line.mp.	8274
11	(virtual not virtual realit:).mp.	283
12	world wide web:.mp.	137
13	worldwide web:.mp.	9
14	www.mp.	33
15	web.mp.	1506
16	web page:.mp.	60
17	webpage:.mp.	12
18	web site:.mp.	396
19	website:.mp.	735
20	exp computer communication networks/	449
21	(computer: adj2 network:).mp.	204
22	(computer-based or computerbased).mp.	581

23	portal?.mp.	1555
24	ehealth.mp.	6
25	e-health.mp.	21
26	"web 2.0".mp.	0
27	semantic web?.mp.	0
28	blog:.mp.	8
29	collabor:.mp.	11320
30	folksonom:.mp.	0
31	mashup:.mp.	0
32	pod cast:.mp.	0
33	podcast:.mp.	0
34	rss feed:.mp.	0
35	really simple syndicat:.mp.	0
36	rss syndicat:.mp.	0
37	(social adj2 bookmark:).mp.	0
38	(social adj2 book-mark:).mp.	0
39	(social adj2 software:).mp.	1
40	(sociable adj2 technolog:).mp.	0
41	(social adj2 technolog:).mp.	6
42	tag cloud:.mp.	0
43	tag????.mp.	546
44	videocast:.mp.	0
45	video-cast:.mp.	0
46	vodcast:.mp.	0
47	vod-cast:.mp.	0
48	web api:.mp.	0
49	(web: adj2 syndicat:).mp.	0
50	webcast:.mp.	2
51	web-cast:.mp.	0
52	web-log:.mp.	1
53	weblog:.mp.	1
54	wiki:.mp.	1
55	social network:.mp.	144
56	(social adj2 utilit:).mp.	6
57	or/1-56	24931
58	(evaluat: adj2 (framework: or frame-work:)).mp.	50

59	(informatic: adj2 evaluat:).mp.	1
60	(evaluat: adj2 guideline?).mp.	216
61	(evaluat: adj2 (process: or protocol: or method? or format? or outline?)).mp.	3856
62	or/58-61	4101
63	57 and 62	559
64	remove duplicates from 63	557

PsycINFO

Run January 5th, 2008

**PsycINFO** 1985 to December Week 3 2007		
**#**	**Search History**	**Results**
1	exp internet/	8523
2	internet:.mp.	11937
3	exp information technology/	2998
4	exp computer-assisted instruction/	7526
5	online.mp.	6940
6	on-line.mp.	2956
7	(virtual not virtual realit:).mp.	2834
8	world wide web:.mp.	1062
9	worldwide web:.mp.	18
10	www.mp.	243
11	web.mp.	7056
12	web page:.mp.	399
13	webpage:.mp.	29
14	web site:.mp.	1621
15	website:.mp.	1577
16	exp computer applications/	32117
17	portal?.mp.	328
18	ehealth.mp.	52
19	e-health.mp.	100
20	"web 2.0".mp.	4
21	semantic web?.mp.	55
22	blog:.mp.	78
23	collabor:.mp.	22616

24	folksonom:.mp.	1
25	mashup:.mp.	0
26	pod cast:.mp.	0
27	podcast:.mp.	11
28	rss feed:.mp.	1
29	really simple syndicat:.mp.	0
30	rss syndicat:.mp.	0
31	(social adj2 bookmark:).mp.	0
32	(social adj2 book-mark:).mp.	0
33	(social adj2 software:).mp.	32
34	(sociable adj2 technolog:).mp.	2
35	(social adj2 technolog:).mp.	389
36	tag cloud:.mp.	0
37	tag????.mp.	960
38	videocast:.mp.	0
39	video-cast:.mp.	0
40	vodcast:.mp.	0
41	vod-cast:.mp.	0
42	web api:.mp.	0
43	(web: adj2 syndicat:).mp.	1
44	webcast:.mp.	27
45	web-cast:.mp.	2
46	web-log:.mp.	15
47	weblog:.mp.	28
48	wiki:.mp.	16
49	social network:.mp.	6923
50	(social adj2 utilit:).mp.	197
51	or/1-50	80639
52	(evaluat: adj2 (framework: or frame-work:)).mp.	553
53	(informatic: adj2 evaluat:).mp.	2
54	(evaluat: adj2 guideline?).mp.	271
55	(evaluat: adj2 (process: or protocol: or method? or format? or outline?)).mp.	5729
56	or/52-55	6477
57	51 and 56	631
58	limit 57 to human	584
59	limit 58 to english	565

60	limit 59 to yr="1990 - 2008"	518
61	remove duplicates from 60	506
62	Exp Evaluation Criteria/ Or Exp Evaluation/ Or Exp Course Evaluation/ Or Exp Program Evaluation/ Or Exp Educational Program Evaluation/	42477
63	exp Models/ or exp Theories/ or exp Methodology/	142573
64	62 and 63	3967
65	51 and 64	264
66	61 or 65	746
67	limit 66 to (human and english language)	720
68	limit 67 to yr="1990 - 2008"	704
69	limit 68 to all journals	504
70	remove duplicates from 69	500

ERIC

Run: January 9th 2008

Search Query #10 (KW=((**evaluat*** within 2 (**framework** * OR **frame-work***)) or (**informatic*** within 2 **evaluat** *) or (**evaluat*** within 2 (**process*** OR **protocol*** OR **method*** OR **format*** OR **outline***)))) and (KW=(**internet*** or **informatic*** or (**computer*** within 2 (**educat** * OR **train***))) or KW=((**online** OR **on-line**) or **virtual*** or ("**world wide web***")) or KW=((" **worldwide web***") or **www** or **webpage***) or KW=(**website** * or **web*** or (**computer*** within 2 **network***)) or KW=("e-**health**" or **ehealth** or ("**web** 2.0")) or KW=(("**semantic web***") or **blog*** or **collabor** *) or KW=(**folksonom*** or **mashup*** or (**podcast*** OR "**pod cast***")) or KW=(("**rss feed***") or (" **really simple syndicat***") or (**social** within 2 (**bookmark*** OR **book-mark***))) or KW=((**social** within 2 **technolog***) or ("**tag cloud***") or **tag***) or KW=(**videocast*** or "video-cast*" or (**vodcast*** OR "vod-cast*")) or KW=((**web*** within 2 **syndicat***) or **webcast*** or **weblog***) or KW=(**wiki*** or ("**social network** *") or (**social** within 2 **utilit***))) **AND KW=(health* OR medic* OR hospital OR hospitals OR clinic* OR patient OR patients)**
