# Supplementary material for: Measuring the Impact of a Moving Target: Towards a Dynamic Framework for Evaluating Collaborative Adaptive Interactive Technologies
Source: J Med Internet Res. 2009 Jun 18;11(2):e20. doi: 10.2196/jmir.1058 (PMC2762807; doi:10.2196/jmir.1058)
Supplement: Supplementary file 2 [file jmir_v11i2e20_app2.pdf]

Appendix B – Search string for 2008 - 2009

Medline

Run: January 30, 2009

Ovid MEDLINE(R) 1950 to January Week 3 2009			
#	Searches	Results	Search Type
1	exp internet/	28198	Advanced
2	internet:.mp.	34631	Advanced
3	exp informatics/	6122	Advanced
4	exp computer-assisted instruction/	6511	Advanced
5	online.mp.	15912	Advanced
6	on-line.mp.	14156	Advanced
7	(virtual not virtual realit:).mp.	11521	Advanced
8	world wide web:.mp.	2288	Advanced
9	worldwide web:.mp.	96	Advanced
10	www.mp.	1094	Advanced
11	web.mp.	21405	Advanced
12	web page:.mp.	723	Advanced
13	webpage:.mp.	73	Advanced
14	web site:.mp.	3510	Advanced
15	website:.mp.	3650	Advanced
16	exp computer communication networks/	38964	Advanced
17	portal?.mp.	53996	Advanced
18	ehealth.mp.	226	Advanced
19	e-health.mp.	439	Advanced
20	"web 2.0".mp.	57	Advanced
21	semantic web?.mp.	123	Advanced
22	blog:.mp.	145	Advanced
23	collabor:.mp.	46973	Advanced
24	folksonom:.mp.	2	Advanced
25	mashup:.mp.	11	Advanced
26	pod cast:.mp.	2	Advanced
27	podcast:.mp.	48	Advanced

28	rss feed:.mp.	13	Advanced
29	really simple syndicat:.mp.	8	Advanced
30	rss syndicat:.mp.	1	Advanced
31	(social adj2 bookmark:).mp.	1	Advanced
32	(social adj2 book-mark:).mp.	0	Advanced
33	(social adj2 software:).mp.	25	Advanced
34	(sociable adj2 technolog:).mp.	1	Advanced
35	(social adj2 technolog:).mp.	224	Advanced
36	tag cloud:.mp.	0	Advanced
37	tag????.mp.	43880	Advanced
38	videocast:.mp.	1	Advanced
39	video-cast:.mp.	1	Advanced
40	(virtual adj2 collabor:).mp.	43	Advanced
41	vodcast:.mp.	1	Advanced
42	vod-cast:.mp.	0	Advanced
43	web api:.mp.	0	Advanced
44	(web adj2 syndicat:).mp.	4	Advanced
45	webcast:.mp.	112	Advanced
46	web-cast:.mp.	0	Advanced
47	web-log:.mp.	24	Advanced
48	weblog:.mp.	18	Advanced
49	wiki:.mp.	90	Advanced
50	social network:.mp.	3355	Advanced
51	(social adj2 utilit:).mp.	75	Advanced
52	or/1-51	241969	Advanced
53	exp Education, Nonprofessional/	162243	Advanced
54	exp Consumer Participation/	23739	Advanced
55	social support/	33767	Advanced
56	((patient? or consumer? or client:) adj2 (decisionmak: or decision-mak:)).mp.	1425	Advanced
57	exp Self Care/	27987	Advanced
58	((self adj2 manag:) or self-manag: or selfmanag:).mp.	4362	Advanced
59	or/53-58	235610	Advanced
60	exp evaluation studies as topic/	727324	Advanced

61	evaluation studies.pt.	110766	Advanced
62	"outcome and process assessment(health care)"/	16761	Advanced
63	"outcome assessment (health care)"/	31077	Advanced
64	"process assessment (health care)"/	2176	Advanced
65	randomized controlled trial.pt.	261036	Advanced
66	evaluation studies.pt.	110766	Advanced
67	qualitative research/	6682	Advanced
68	user-computer interface/	16426	Advanced
69	usability.mp.	1765	Advanced
70	useability.mp.	22	Advanced
71	accessibility.mp.	46652	Advanced
72	sociability.mp.	642	Advanced
73	log file?.mp.	90	Advanced
74	readability.mp.	838	Advanced
75	or/60-74	1126144	Advanced
76	52 and 59 and 75	3120	Advanced
77	limit 76 to (humans and english language)	2882	Advanced
78	limit 77 to yr="2008 - 2009"	337	Advanced
79	2008:.ed.	719226	Advanced
80	2009:.ed.	28995	Advanced
81	80 or 79	748221	Advanced
82	81 and 77	466	Advanced
83	78 or 82	466	Advanced
84	remove duplicates from 83	463	Advanced
85	from 84 keep 1-463	463	Advanced

CINAHL Search strategy for conversion to EbscoHost

Run: January 30, 2009

Ovid MEDLINE(R) 1950 to January Week 3 2009			
#	Searches	Results	Search Type
1	exp internet/	28198	Advanced
2	internet:.tw.	14632	Advanced
3	exp informatics/	6122	Advanced

4	exp computer-assisted instruction/	6511	Advanced
5	online.tw.	11818	Advanced
6	on-line.tw.	14140	Advanced
7	(virtual not virtual realit:).tw.	11521	Advanced
8	world wide web:.tw.	2288	Advanced
9	worldwide web:.tw.	96	Advanced
10	www.tw.	1093	Advanced
11	web.tw.	21256	Advanced
12	web page:.tw.	723	Advanced
13	webpage:.tw.	73	Advanced
14	web site:.tw.	3510	Advanced
15	website:.tw.	3649	Advanced
16	exp computer communication networks/	38964	Advanced
17	portal?.tw.	46529	Advanced
18	ehealth.tw.	225	Advanced
19	e-health.tw.	439	Advanced
20	"web 2.0".tw.	57	Advanced
21	semantic web?.tw.	123	Advanced
22	blog:.tw.	136	Advanced
23	collabor:.tw.	46860	Advanced
24	folksonom:.tw.	2	Advanced
25	mashup:.tw.	11	Advanced
26	pod cast:.tw.	2	Advanced
27	podcast:.tw.	48	Advanced
28	rss feed:.tw.	13	Advanced
29	really simple syndicat:.tw.	8	Advanced
30	rss syndicat:.tw.	1	Advanced
31	(social adj2 bookmark:).tw.	1	Advanced
32	(social adj2 book-mark:).tw.	0	Advanced
33	(social adj2 software:).tw.	25	Advanced
34	(sociable adj2 technolog:).tw.	1	Advanced
35	(social adj2 technolog:).tw.	224	Advanced
36	tag cloud:.tw.	0	Advanced
37	tag????.tw.	37892	Advanced

38	videocast:.tw.	1	Advanced
39	video-cast:.tw.	1	Advanced
40	vodcast:.tw.	1	Advanced
41	vod-cast:.tw.	0	Advanced
42	web api:.tw.	0	Advanced
43	(web: adj2 syndicat:).tw.	4	Advanced
44	webcast:.tw.	112	Advanced
45	web-cast:.tw.	0	Advanced
46	web-log:.tw.	24	Advanced
47	weblog:.tw.	18	Advanced
48	wiki:.tw.	89	Advanced
49	social network:.tw.	3355	Advanced
50	(social adj2 utilit:).tw.	75	Advanced
51	or/1-50	226702	Advanced
52	exp Education, Nonprofessional/	162243	Advanced
53	exp Consumer Participation/	23739	Advanced
54	social support/	33767	Advanced
55	((patient? or consumer? or client:) adj2 (decisionmak: or decision-mak:)).tw.	1425	Advanced
56	exp Self Care/	27987	Advanced
57	((self adj2 manag:) or self-manag: or selfmanag:).tw.	4362	Advanced
58	or/52-57	235610	Advanced
59	exp evaluation research/	727324	Advanced
60	(evaluat: adj1 (study or studies)).tw.	32976	Advanced
61	"outcomes (health care)"/	0	Advanced
62	outcome assessment/	0	Advanced
63	outcomes research/	31077	Advanced
64	"process assessment (health care)"/	2176	Advanced
65	quality of care research/	0	Advanced
66	health services research/	24465	Advanced
67	exp clinical trials/	0	Advanced
68	qualitative research/	6682	Advanced
69	user-computer interface/	16426	Advanced
70	usability.tw.	1765	Advanced

71	useability.tw.	22	Advanced
72	accessibility.tw.	14751	Advanced
73	sociability.tw.	642	Advanced
74	log file?.tw.	90	Advanced
75	readability.tw.	838	Advanced
76	or/59-75	834990	Advanced
77	51 and 58 and 76	2538	Advanced
78	limit 77 to english	2458	Advanced
79	limit 78 to yr="1990 - 2007"	2133	Advanced
80	limit 79 to journal article	2088	Advanced
81	remove duplicates from 80	2085	Advanced

BREAKDOWN of CONVERSION

eHealth/Internet terms - TI

MH computer communication networks+
MH computer-assisted instruction+
MH informatics+
MH internet+
TI "web 2.0"
TI (sociable N2 technolog*)
TI (social N2 bookmark*)
TI (social N2 book-mark*)
TI (social N2 software*)
TI (social N2 technolog*)
TI (social N2 utilit*)
TI virtual
TI (web* N2 syndicat*)
TI “e-health”
TI blog*
TI collabor*
TI ehealth

TI folksonom*
TI Internet*
TI mashup*
TI online
TI “on-line”
TI pod cast* → confounded search engine
TI podcast*
TI Portal*
TI (really simple syndicat*)
TI (rss feed*)
TI (rss syndicat*)
TI (semantic web*)
TI (social network*)
TI (tag cloud*)
TI (tag or tagging or tagged)
TI videocast*
TI (video-cast*)
TI vodcast*
TI (vod-cast*)
TI (web api*)
TI web
TI (web page*)
TI (web site*)
TI webcast*
TI (web-cast*)
TI weblog*
TI (web-log*)
TI webpage
TI Website*
TI wiki*
TI (world wide web*)
TI (worldwide web*)
TI www

TI ("web 2.0" OR (sociable N2 technolog*) OR (social N2 bookmark*) OR (social N2 book-mark*) OR (social N2 software*) OR (social N2 technolog*) OR (social N2 utilit*) OR virtual OR (web* N2 syndicat*) OR “e-health” OR blog* OR collabor* OR ehealth OR folksonom* OR Internet* OR mashup* OR online OR “on-line” OR pod cast* → confounded search engine OR podcast* OR Portal* OR (really simple syndicat*) OR (rss feed*) OR (rss syndicat*) OR (semantic web*) OR (social network*) OR (tag cloud*) OR (tag or tagging or tagged) OR videocast* OR (video-cast*) OR vodcast* OR (vod-cast*) OR (web api*) OR web OR (web page*) OR (web site*) OR webcast* OR (web-cast*) OR weblog* OR (web-log*) OR webpage OR Website* OR wiki* OR (world wide web*) OR (worldwide web*) OR www)

AB ("web 2.0" OR (sociable N2 technolog*) OR (social N2 bookmark*) OR (social N2 book-mark*) OR (social N2 software*) OR (social N2 technolog*) OR (social N2 utilit*) OR virtual OR (web* N2 syndicat*) OR “e-health” OR blog* OR collabor* OR ehealth OR folksonom* OR Internet* OR mashup* OR online OR “on-line” OR pod cast* → confounded search engine OR podcast* OR Portal* OR (really simple syndicat*) OR (rss feed*) OR (rss syndicat*) OR (semantic web*) OR (social network*) OR (tag cloud*) OR (tag or tagging or tagged) OR videocast* OR (video-cast*) OR vodcast* OR (vod-cast*) OR (web api*) OR web OR (web page*) OR (web site*) OR webcast* OR (web-cast*) OR weblog* OR (web-log*) OR webpage OR Website* OR wiki* OR (world wide web*) OR (worldwide web*) OR www)

Patient ed/social support

exp Education, Nonprofessional/
exp Consumer Participation/
social support/
((patient? or consumer? or client:) adj2 (decisionmak: or decision-mak:)).tw.
exp Self Care/
((self adj2 manag:) or self-manag: or selfmanag:).tw.

MH Education, Nonprofessional+
MH Consumer Participation+
MH Self care+
MH Social support

TI ((patient* N2 decisionmak*) OR (patient* N2 decision-mak*) OR (consumer* N2 decisionmak*) OR (consumer* N2 decision-mak*) OR (client* N2 decisionmak*) OR (client* N2 decision-mak*))

TI (self N2 manag*) or self-manag* or selfmanag*
AB (self N2 manag*) or self-manag* or selfmanag*

Evaluation segment

exp evaluation research/
(evaluat: adj1 (study or studies)).tw.
"outcomes (health care)"/
outcome assessment/
outcomes research/
"process assessment (health care)"/
quality of care research/
health services research/
exp clinical trials/
qualitative research/
user-computer interface/
usability.tw.
useability.tw.

accessibility.tw.
sociability.tw.
log file?.tw.
readability.tw.

MH evaluation research+
MH "outcomes (health care)"
MH outcome assessment
MH outcomes research
MH "process assessment (health care)"
MH quality of care research
MH health services research
MH clinical trials+
MH qualitative research
MH user-computer interface

MH (evaluation research+ OR "outcomes (health care)" OR outcome assessment OR outcomes research OR "process assessment (health care)" OR quality of care research OR health services research OR clinical trials+ OR qualitative research OR user-computer interface)

TI (evaluat* N1 (study or studies)) OR usability OR useability OR accessibility OR sociability OR log file* OR readability

AB (evaluat* N1 (study or studies)) OR usability OR useability OR accessibility OR sociability OR log file* OR readability

Converted search strategy run over CINAHL on EBSCO Host

Friday, January 30, 2009 3:25:41 PM

#	Query	Limiters/Expanders	Last Run Via	Results
S26	S9 and S20 and S24	**Limiters** - Publication Year from: 2008-2009<br>**Search modes** - Boolean/Phrase	**Interface** - EBSCOhost<br>**Search Screen** - Advanced Search<br>**Database** - CINAHL	389
S25	S9 and S20 and S24	**Search modes** - Boolean/Phrase	**Interface** - EBSCOhost<br>**Search Screen** - Advanced	3534

			Search<br>**Database** - CINAHL	
S24	S21 or S22 or S23	**Search modes** - Boolean/Phrase	**Interface** - EBSCOhost<br>**Search Screen** - Advanced Search<br>**Database** - CINAHL	203815
S23	AB (evaluat* N1 (study or studies)) OR usability OR useability OR accessibility OR sociability OR log file* OR readability	**Search modes** - Boolean/Phrase	**Interface** - EBSCOhost<br>**Search Screen** - Advanced Search<br>**Database** - CINAHL	Display
S22	TI (evaluat* N1 (study or studies)) OR usability OR useability OR accessibility OR sociability OR log file* OR readability	**Search modes** - Boolean/Phrase	**Interface** - EBSCOhost<br>**Search Screen** - Advanced Search<br>**Database** - CINAHL	Display
S21	MH evaluation research+ OR "outcomes (health care)" OR outcome assessment OR outcomes research OR "process assessment (health care)" OR quality of care research OR health services research OR clinical trials+ OR qualitative research OR user-computer interface	**Search modes** - Boolean/Phrase	**Interface** - EBSCOhost<br>**Search Screen** - Advanced Search<br>**Database** - CINAHL	Display
S20	S14 or S19	**Search modes** - Boolean/Phrase	**Interface** - EBSCOhost<br>**Search Screen** - Advanced Search<br>**Database** - CINAHL	98840
S19	S15 or S16 or S17 or S18	**Search modes** - Boolean/Phrase	**Interface** - EBSCOhost<br>**Search Screen** - Advanced Search<br>**Database** - CINAHL	Display
S18	AB (self N2 manag*) or self-manag* or selfmanag*	**Search modes** - Boolean/Phrase	**Interface** - EBSCOhost<br>**Search Screen** - Advanced Search<br>**Database** - CINAHL	Display
S17	TI (self N2 manag*) or self-manag* or selfmanag*	**Search modes** - Boolean/Phrase	**Interface** - EBSCOhost<br>**Search Screen** - Advanced Search<br>**Database** - CINAHL	Display
S16	AB (patient* N2 decisionmak*) OR (patient* N2 decision-mak*) OR (consumer* N2 decisionmak*) OR (consumer* N2 decision-mak*) OR (client* N2 decisionmak*) OR (client* N2 decision-mak*)	**Search modes** - Boolean/Phrase	**Interface** - EBSCOhost<br>**Search Screen** - Advanced Search<br>**Database** - CINAHL	Display
S15	TI (patient* N2 decisionmak*) OR (patient* N2 decision-mak*) OR (consumer* N2 decisionmak*) OR (consumer* N2 decision-mak*) OR (client* N2 decisionmak*) OR (client* N2 decision-mak*)	**Search modes** - Boolean/Phrase	**Interface** - EBSCOhost<br>**Search Screen** - Advanced Search<br>**Database** - CINAHL	Display

S14	S10 or S11 or S12 or S13	**Search modes** - Boolean/Phrase	**Interface** - EBSCOhost<br>**Search Screen** - Advanced Search<br>**Database** - CINAHL	Display
S13	MH Support, Psychosocial	**Search modes** - Boolean/Phrase	**Interface** - EBSCOhost<br>**Search Screen** - Advanced Search<br>**Database** - CINAHL	Display
S12	MH Self care+	**Search modes** - Boolean/Phrase	**Interface** - EBSCOhost<br>**Search Screen** - Advanced Search<br>**Database** - CINAHL	Display
S11	MH Consumer Participation+	**Search modes** - Boolean/Phrase	**Interface** - EBSCOhost<br>**Search Screen** - Advanced Search<br>**Database** - CINAHL	Display
S10	MH Education, Nonprofessional+	**Search modes** - Boolean/Phrase	**Interface** - EBSCOhost<br>**Search Screen** - Advanced Search<br>**Database** - CINAHL	Display
S9	S5 or S8	**Search modes** - Boolean/Phrase	**Interface** - EBSCOhost<br>**Search Screen** - Advanced Search<br>**Database** - CINAHL	229883
S8	S6 or S7	**Search modes** - Boolean/Phrase	**Interface** - EBSCOhost<br>**Search Screen** - Advanced Search<br>**Database** - CINAHL	Display
S7	AB ("web 2.0" OR (sociable N2 technolog*) OR (social N2 bookmark*) OR (social N2 book-mark*) OR (social N2 software*) OR (social N2 technolog*) OR (social N2 utilit*) OR virtual OR (web* N2 syndicat*) OR "e-health" OR blog* OR collabor* OR ehealth OR folksonom* OR Internet* OR mashup* OR online OR "on-line" OR pod confounded search engine OR podcast* OR Portal* OR (really simple◊ cast* syndicat*) OR (rss feed*) OR (rss syndicat*) OR (semantic web*) OR (social network*) OR (tag cloud*) OR (tag or tagging or tagged) OR videocast* OR (video-cast*) OR vodcast* OR (vod-cast*) OR (web api*) OR web OR (web page*) OR (web site*) OR webcast* OR (web-cast*) OR weblog* OR (web-log*) OR webpage OR Website* OR wiki* OR (world wide web*) OR (worldwide web*) OR www)	**Search modes** - Boolean/Phrase	**Interface** - EBSCOhost<br>**Search Screen** - Advanced Search<br>**Database** - CINAHL	Display
S6	TI ("web 2.0" OR (sociable	**Search modes** -	**Interface** - EBSCOhost	Display

	N2 technolog*) OR (social N2 bookmark*) OR (social N2 book-mark*) OR (social N2 software*) OR (social N2 technolog*) OR (social N2 utilit*) OR virtual OR (web* N2 syndicat*) OR "e-health" OR blog* OR collabor* OR ehealth OR folksonom* OR Internet* OR mashup* OR online OR "on-line" OR pod confounded search engine OR podcast* OR Portal* OR (really simple◊ cast* syndicat*) OR (rss feed*) OR (rss syndicat*) OR (semantic web*) OR (social network*) OR (tag cloud*) OR (tag or tagging or tagged) OR videocast* OR (video-cast*) OR vodcast* OR (vod-cast*) OR (web api*) OR web OR (web page*) OR (web site*) OR webcast* OR (web-cast*) OR weblog* OR (web-log*) OR webpage OR Website* OR wiki* OR (world wide web*) OR (worldwide web*) OR www)	Boolean/Phrase	**Search Screen** - Advanced Search<br>**Database** - CINAHL	
S5	S1 or S2 or S3 or S4	**Search modes** - Boolean/Phrase	**Interface** - EBSCOhost<br>**Search Screen** - Advanced Search<br>**Database** - CINAHL	Display
S4	MH internet+	**Search modes** - Boolean/Phrase	**Interface** - EBSCOhost<br>**Search Screen** - Advanced Search<br>**Database** - CINAHL	Display
S3	MH informatics+	**Search modes** - Boolean/Phrase	**Interface** - EBSCOhost<br>**Search Screen** - Advanced Search<br>**Database** - CINAHL	Display
S2	MH "Computer Assisted Instruction"	**Search modes** - Boolean/Phrase	**Interface** - EBSCOhost<br>**Search Screen** - Advanced Search<br>**Database** - CINAHL	Display
S1	MH computer communication networks+	**Search modes** - Boolean/Phrase	**Interface** - EBSCOhost<br>**Search Screen** - Advanced Search<br>**Database** - CINAHL	Display

All EBMR (Cochrane)

Run: January 30, 2009

All EBM Reviews - Cochrane DSR, ACP Journal Club, DARE, CCTR, CMR, HTA, and NHSEED			
#	Searches	Results	Search Type
1	exp internet/	521	Advanced
2	internet:.mp.	1857	Advanced
3	exp informatics/	42	Advanced
4	exp computer-assisted instruction/	467	Advanced
5	online.mp.	6260	Advanced
6	on-line.mp.	8986	Advanced
7	(virtual not virtual realit:).mp.	313	Advanced
8	world wide web:.mp.	145	Advanced
9	worldwide web:.mp.	9	Advanced
10	www.mp.	37	Advanced
11	web.mp.	1887	Advanced
12	web page:.mp.	68	Advanced
13	webpage:.mp.	13	Advanced
14	web site:.mp.	459	Advanced
15	website:.mp.	972	Advanced
16	exp computer communication networks/	581	Advanced
17	portal?.mp.	1662	Advanced
18	ehealth.mp.	18	Advanced
19	e-health.mp.	22	Advanced
20	"web 2.0".mp.	0	Advanced
21	semantic web?.mp.	0	Advanced
22	blog:.mp.	12	Advanced
23	collabor:.mp.	12051	Advanced
24	folksonom:.mp.	0	Advanced

25	mashup:.mp.	0	Advanced
26	pod cast:.mp.	0	Advanced
27	podcast:.mp.	0	Advanced
28	rss feed:.mp.	0	Advanced
29	really simple syndicat:.mp.	0	Advanced
30	rss syndicat:.mp.	0	Advanced
31	(social adj2 bookmark:).mp.	0	Advanced
32	(social adj2 book-mark:).mp.	0	Advanced
33	(social adj2 software:).mp.	1	Advanced
34	(sociable adj2 technolog:).mp.	0	Advanced
35	(social adj2 technolog:).mp.	8	Advanced
36	tag cloud:.mp.	0	Advanced
37	tag????.mp.	594	Advanced
38	videocast:.mp.	0	Advanced
39	video-cast:.mp.	1	Advanced
40	(virtual adj2 collabor:).mp.	1	Advanced
41	vodcast:.mp.	0	Advanced
42	vod-cast:.mp.	0	Advanced
43	web api:.mp.	0	Advanced
44	(web adj2 syndicat:).mp.	0	Advanced
45	webcast:.mp.	2	Advanced
46	web-cast:.mp.	0	Advanced
47	web-log:.mp.	2	Advanced
48	weblog:.mp.	2	Advanced
49	wiki:.mp.	3	Advanced
50	social network:.mp.	160	Advanced
51	(social adj2 utilit:).mp.	6	Advanced
52	or/1-51	28283	Advanced
53	exp Education, Nonprofessional/	4136	Advanced
54	exp Consumer Participation/	637	Advanced
55	social support/	1310	Advanced
56	((patient? or consumer? or client:) adj2 (decisionmak: or decision-mak:)).mp.	272	Advanced
57	exp Self Care/	2192	Advanced

58	((self adj2 manag:) or self-manag: or selfmanag:).mp.	1231	Advanced
59	or/53-58	8915	Advanced
60	exp evaluation studies as topic/	0	Advanced
61	evaluation studies.pt.	1011	Advanced
62	"outcome and process assessment(health care)"/	1293	Advanced
63	"outcome assessment (health care)"/	2432	Advanced
64	"process assessment (health care)"/	85	Advanced
65	randomized controlled trial.pt.	249900	Advanced
66	evaluation studies.pt.	1011	Advanced
67	qualitative research/	94	Advanced
68	user-computer interface/	446	Advanced
69	usability.mp.	88	Advanced
70	useability.mp.	2	Advanced
71	accessibility.mp.	793	Advanced
72	sociability.mp.	40	Advanced
73	log file?.mp.	5	Advanced
74	readability.mp.	206	Advanced
75	or/60-74	251888	Advanced
76	52 and 59 and 75	451	Advanced
77	limit 76 to (humans and english language) [Limit not valid in CDSR,ACP Journal Club,DARE,CCTR,CLCMR; records were retained]	451	Advanced
78	limit 77 to yr="2008 - 2009" [Limit not valid in DARE; records were retained]	53	Advanced

PsycINFO

Run: January 30, 2009

PsycINFO 2002 to January Week 4 2009			
#	Searches	Results	Search Type
1	exp internet/	9209	Advanced
2	internet:.mp.	12319	Advanced
3	exp information technology/	2107	Advanced
4	exp computer-assisted instruction/	3723	Advanced
5	online.mp.	8033	Advanced
6	on-line.mp.	1668	Advanced
7	(virtual not virtual realit:).mp.	2368	Advanced
8	world wide web:.mp.	608	Advanced
9	worldwide web:.mp.	15	Advanced
10	www.mp.	111	Advanced
11	web.mp.	6928	Advanced
12	web page:.mp.	372	Advanced
13	webpage:.mp.	28	Advanced
14	web site:.mp.	1629	Advanced
15	website:.mp.	2020	Advanced
16	exp computer applications/	14804	Advanced
17	portal?.mp.	296	Advanced
18	ehealth.mp.	60	Advanced
19	e-health.mp.	113	Advanced
20	"web 2.0".mp.	15	Advanced
21	semantic web?.mp.	75	Advanced
22	blog:.mp.	185	Advanced
23	collabor:.mp.	15153	Advanced
24	folksonom:.mp.	2	Advanced
25	mashup:.mp.	0	Advanced
26	pod cast:.mp.	0	Advanced
27	podcast:.mp.	33	Advanced
28	rss feed:.mp.	3	Advanced

29	really simple syndicat:.mp.	0	Advanced
30	rss syndicat:.mp.	0	Advanced
31	(social adj2 bookmark:).mp.	4	Advanced
32	(social adj2 book-mark:).mp.	0	Advanced
33	(social adj2 software:).mp.	29	Advanced
34	(sociable adj2 technolog:).mp.	1	Advanced
35	(social adj2 technolog:).mp.	218	Advanced
36	tag cloud:.mp.	1	Advanced
37	tag????.mp.	742	Advanced
38	videocast:.mp.	0	Advanced
39	video-cast:.mp.	0	Advanced
40	vodcast:.mp.	0	Advanced
41	vod-cast:.mp.	0	Advanced
42	web api:.mp.	0	Advanced
43	(web: adj2 syndicat:).mp.	1	Advanced
44	webcast:.mp.	28	Advanced
45	web-cast:.mp.	3	Advanced
46	web-log:.mp.	23	Advanced
47	weblog:.mp.	48	Advanced
48	wiki:.mp.	51	Advanced
49	social network:.mp.	3816	Advanced
50	(social adj2 utilit:).mp.	101	Advanced
51	or/1-50	52317	Advanced
52	Education/	5743	Advanced
53	client education/	703	Advanced
54	consumer education/	37	Advanced
55	exp nontraditional education/	275	Advanced
56	exp client participation/	525	Advanced
57	social support/	6715	Advanced
58	((patient? or consumer? or client:) adj2 (decisionmak: or decision-mak:)).mp.	443	Advanced
59	exp Self Care/	644	Advanced
60	((self adj2 manag:) or self-manag: or selfmanag:).mp.	2124	Advanced
61	or/52-60	16871	Advanced

62	evaluation/	4255	Advanced
63	evaluation criteria/	413	Advanced
64	exp Treatment Effectiveness Evaluation/	5069	Advanced
65	human computer interaction/	2369	Advanced
66	(evaluat: adj1 (study or studies)).mp.	4090	Advanced
67	(outcome? adj1 assess:).mp.	725	Advanced
68	(process?? adj1 assess:).mp.	824	Advanced
69	qualitative research/	1638	Advanced
70	usability.mp.	955	Advanced
71	useability.mp.	2	Advanced
72	accessibility.mp.	2181	Advanced
73	sociability.mp.	571	Advanced
74	log file?.mp.	53	Advanced
75	readability.mp.	359	Advanced
76	(random: adj7 (trial? or study or studies)).mp.	13797	Advanced
77	(clinical adj7 (trial? or study or studies)).mp.	15840	Advanced
78	or/62-77	45985	Advanced
79	51 and 61 and 78	192	Advanced
80	limit 79 to (human and english language)	181	Advanced
81	limit 80 to yr="1990 - 2008"	181	Advanced
82	limit 81 to all journals	141	Advanced
83	limit 82 to yr="2008 - 2009"	17	Advanced
84	2008:.up.	148816	Advanced
85	2009:.up.	9953	Advanced
86	84 or 85	158769	Advanced
87	86 and 82	30	Advanced
88	87 or 83	30	Advanced

ERIC

Run:

(KW=((**evaluat*** within 2 (**framework*** OR **frame-work***)) or (**informatic*** within 2 **evaluat***) or (**evaluat*** within 2 (**process*** OR **protocol*** OR **method*** OR **format*** OR **outline***)))) and (KW=(**internet*** or **informatic*** or (**computer*** within 2 (**educat*** OR **train***))) or KW=((**online** OR **on-line**) or **virtual*** or (" **world wide web***")) or KW=(("**worldwide web***") or **www** or **webpage***) or KW=(**website*** or **web*** or (**computer*** within 2 **network***)) or KW=("e-**health** " or **ehealth** or ("**web** 2.0")) or KW=(("**semantic web***") or **blog*** or **collabor***) or KW=(**folksonom*** or **mashup*** or (**podcast*** OR "**pod cast** *")) or KW=(("**rss feed***") or ("**really simple syndicat***") or (**social** within 2 (**bookmark*** OR **book-mark***))) or KW=((**social** within 2 **technolog***) or (" **tag cloud***") or **tag***) or KW=(**videocast*** or "video-cast*" or (**vodcast*** OR "vod-cast*")) or KW=((**web*** within 2 **syndicat***) or **webcast*** or **weblog** *) or KW=(**wiki*** or ("**social network***") or (**social** within 2 **utilit***))) AND **KW=(health* OR medic* OR patient OR patients)**
91 results found in ERIC
Date Range: Earliest to 2008
Limited to: Published Works Only; Journal Articles Only; English Only

Social Sciences Abstracts OR Social Sciences Citation Index

Run:

Search Query #6 (((KW=((**evaluat*** within 2 (**framework*** OR **frame-work***)) or (**informatic*** within 2 **evaluat***) or (**evaluat*** within 2 (**process*** OR **protocol*** OR **method*** OR **format*** OR **outline***)))) and (KW=(**internet*** or **informatic*** or (**computer*** within 2 (**educat*** OR **train***))) or KW=((**online** OR **on-line**) or **virtual*** or ("**world wide web***")) or KW=(("**worldwide web***") or **www** or **webpage***) or KW=(**website*** or **web*** or (**computer*** within 2 **network***)) or KW=("e-health" or **ehealth** or ("**web** 2.0")) or KW=(("**semantic web***") or **blog*** or **collabor***) or KW=(**folksonom*** or **mashup*** or (**podcast*** OR "**pod cast***")) or KW=(("**rss feed***") or ("**really simple syndicat***") or (**social** within 2 (**bookmark*** OR **book-mark***))) or KW=((**social** within 2 **technolog***) or ("**tag cloud***") or **tag***) or KW=(**videocast*** or "video-cast*" or (**vodcast*** OR "vod-cast*")) or KW=((**web*** within 2 **syndicat***) or **webcast*** or **weblog***) or KW=(**wiki*** or ("**social network***") or (**social** within 2 **utilit***))))

Scopus

Run: February 6th, 2009

((((online* OR on-line) OR ("web page*" OR webpage*) OR (website* OR "web site*") OR internet* OR (computer* W/2 network*) OR ("computer* 1,447 Scopus

system*") OR folksonom* OR mashup* OR ("pod cast*" OR podcast*) OR "rss feed*" OR "really simple syndicat*" OR "social w/2 bookmark*" OR "social w/2 book-mark*" OR "tag cloud*" OR videocast* OR video-cast* OR "web w/2 syndicat*" OR webcast* OR "web-cast*" OR weblog* OR "web-log*" OR wiki* OR "social w/2 utilit*") AND (comput* OR internet* OR wireless OR mobil* OR "web-based*" OR "webbased" OR cyber*) AND SUBJAREA(mult OR agri OR bioc OR immu OR neur OR phar OR mult OR medi OR nurs OR vete OR dent OR heal OR mult OR arts OR busi OR deci OR econ OR psyc OR soci) AND PUBYEAR AFT 1989) AND (((evaluat* W/2 framework*) OR (evaluat* W/2 frame-work*) OR (informatic* W/2 evaluat*) OR (evaluat* W/2 guideline*) OR (evaluat* W/2 process*) OR (evaluat* W/2 protocol*) OR (evaluat* W/2 method*) OR (evaluat* W/2 format*) OR (evaluat* W/2 outline*)) AND SUBJAREA(mult OR agri OR bioc OR immu OR neur OR phar OR mult OR medi OR nurs OR vete OR dent OR heal OR mult OR arts OR busi OR deci OR econ OR psyc OR soci) AND PUBYEAR AFT 1989)) AND DOCTYPE(ar OR re) AND SUBJAREA(mult OR medi OR nurs OR vete OR dent OR heal OR mult OR arts OR busi OR deci OR econ OR psyc OR soci) AND PUBYEAR AFT 1992 AND (LIMIT-TO(PUBYEAR, 2009) OR LIMIT-TO(PUBYEAR, 2008)) AND (LIMIT-TO(LANGUAGE, "English"))
