# Supplementary figures and images for: Measuring the Impact of a Moving Target: Towards a Dynamic Framework for Evaluating Collaborative Adaptive Interactive Technologies
Source: J Med Internet Res. 2009 Jun 18;11(2):e20. doi: 10.2196/jmir.1058 (PMC2762807; doi:10.2196/jmir.1058)

Appendix C - QUOROM statement flow diagram for Literature Search 2007 and prior

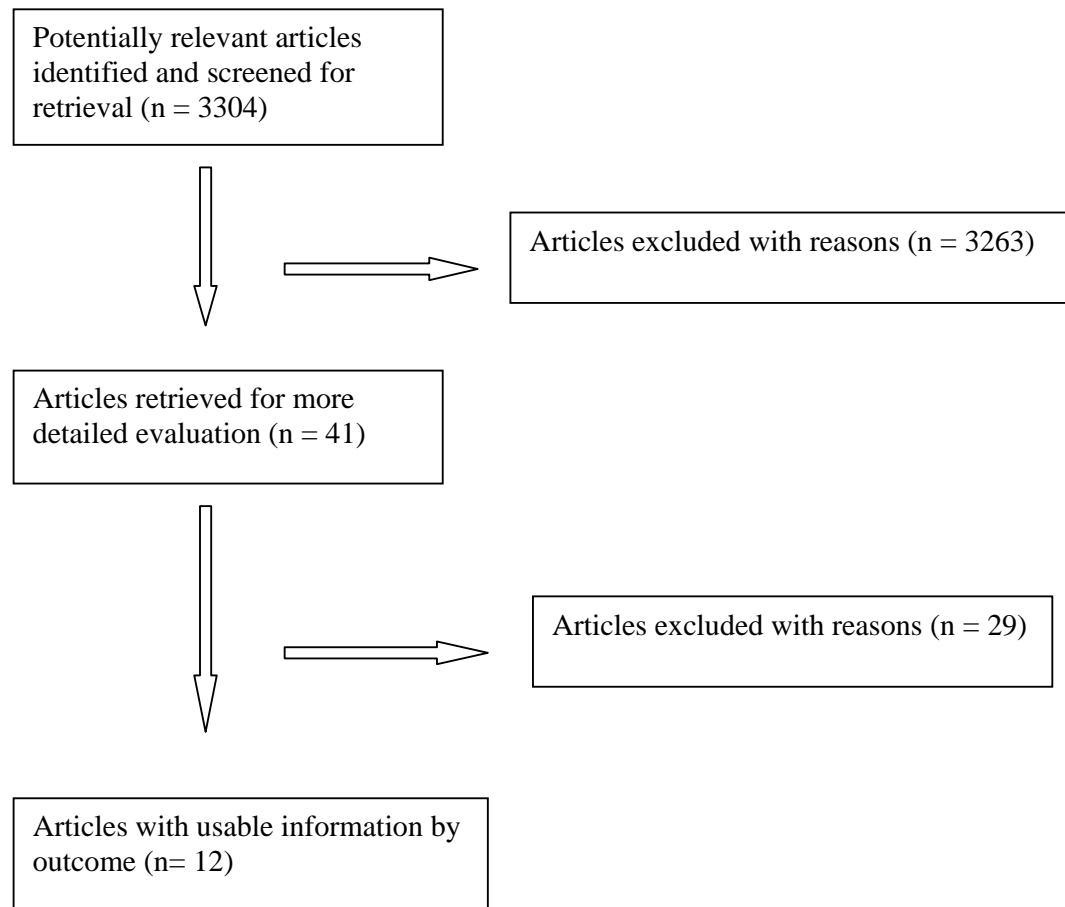

Supplement: Supplementary file 3 [file jmir_v11i2e20_app3.pdf]

Appendix D - QUOROM statement flow diagram for Literature Search 2008 and 2009

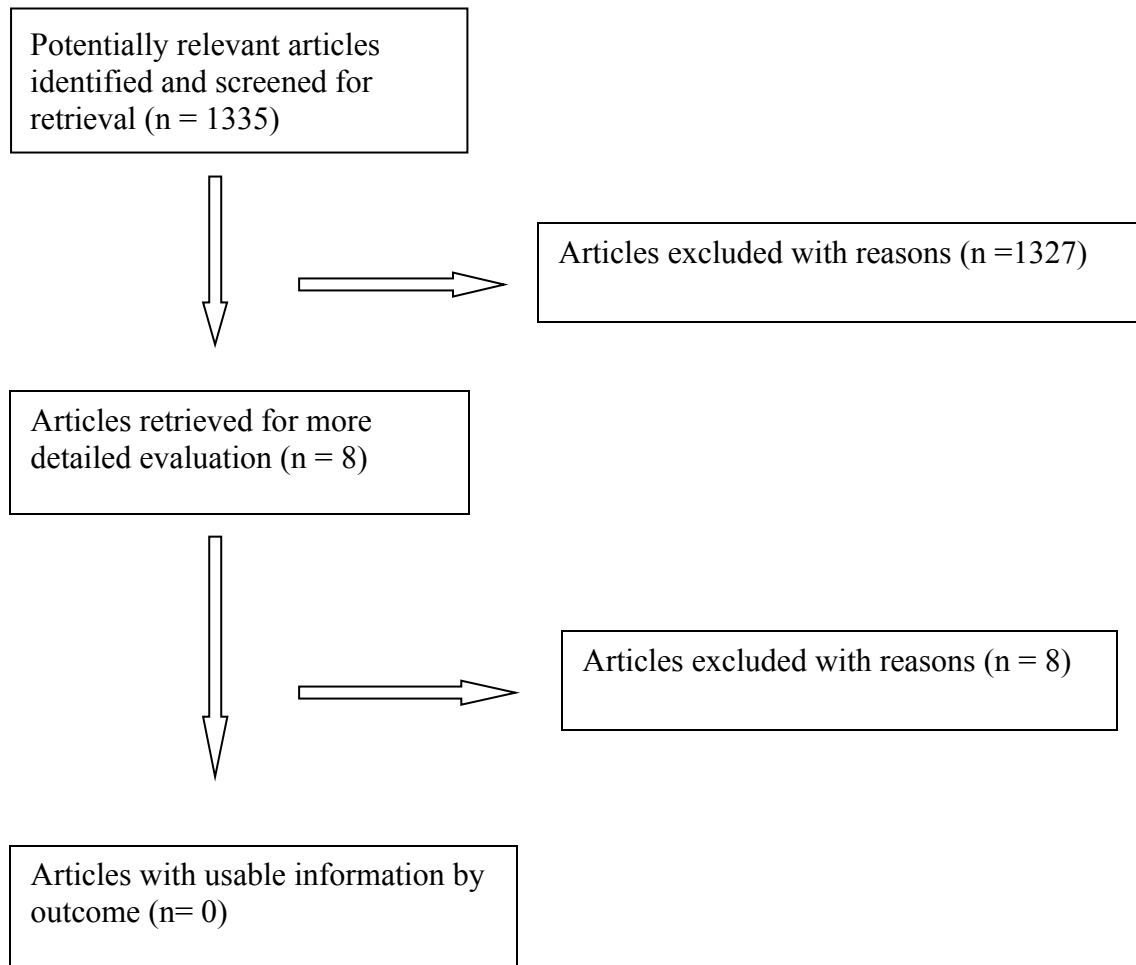

Supplement: Supplementary file 4 [file jmir_v11i2e20_app4.pdf]
